# SBT (Composed of *Panax ginseng* and *Aconitum carmichaeli*) and Stigmasterol Enhances Nitric Oxide Production and Exerts Curative Properties as a Potential Anti-Oxidant and Immunity-Enhancing Agent

**DOI:** 10.3390/antiox11020199

**Published:** 2022-01-20

**Authors:** Na-Ra Han, Kyeoung-Cheol Kim, Ju-Sung Kim, Hi-Joon Park, Seong-Gyu Ko, Phil-Dong Moon

**Affiliations:** 1 College of Korean Medicine, Kyung Hee University, Seoul 02447, Korea; nrhan@khu.ac.kr; 2 Korean Medicine-Based Drug Repositioning Cancer Research Center, College of Korean Medicine, Kyung Hee University, Seoul 02447, Korea; epiko@khu.ac.kr; 3 Majors in Plant Resource and Environment, College of Agriculture & Life Sciences, SARI, Jeju National University, Jeju 63243, Korea; cheolst@jejunu.ac.kr (K.-C.K.); aha2011@jejunu.ac.kr (J.-S.K.); 4 Department of Anatomy & Information Sciences, College of Korean Medicine, Kyung Hee University, Seoul 02447, Korea; acufind@khu.ac.kr; 5 Department of Preventive Medicine, College of Korean Medicine, Kyung Hee University, Seoul 02447, Korea; 6 Center for Converging Humanities, Kyung Hee University, Seoul 02447, Korea

**Keywords:** stigmasterol, nitric oxide, heme oxygenase-1, NK cell cytotoxic activity, cyclophosphamide, superoxide dismutase

## Abstract

Immune dysregulation is a risk factor for several diseases, including infectious diseases. Immunostimulatory agents have been used for the treatment of immune dysregulation, but deleterious adverse effects have been reported. The present study aims to establish the anti-oxidant and immunity-enhancing effects of Sambu-Tang (SBT), composed of *Panax ginseng* and *Aconitum carmichaeli*, and stigmasterol (Stig), an active compound of SBT. Immune-related factors were analyzed in RAW264.7 macrophage cells, mouse primary splenocytes, and the serum and spleen of cyclophosphamide-induced immunosuppressed mice. Results showed that the production levels of nitric oxide (NO) and expression levels of inducible NO synthase and heme oxygenase-1 were increased following SBT or Stig treatment in RAW264.7 cells. SBT or Stig increased the production levels of G-CSF, IFN-γ, IL-12, IL-2, IL-6, and TNF-α and induced the activation of NF-κB in RAW264.7 cells. SBT or Stig promoted splenic lymphocyte proliferation and increased splenic NK cell cytotoxic activity. In addition, SBT or Stig enhanced the levels of IFN-γ, IL-12, IL-2, IL-6, or TNF-α in the serum and spleen of the immunosuppressed mice. SBT or Stig increased the superoxide dismutase activity in the spleen. Collectively, SBT and Stig possess anti-oxidant and immunomodulatory activities, so they may be considered effective natural compounds for the treatment of various symptoms caused by immune dysregulation.

## 1. Introduction

The immune system plays a critical role in controlling infections, healing injuries, and restoring homeostasis [1]. The anti-oxidant defense systems are important for the normal function of the immune system [2]. Immune dysregulation is a risk factor for several diseases, including autoimmune disease and infectious disease [3,4]. Patients with immunodeficiency disorders usually have increased frequency, severity, or persistence of infections [5]. Immunostimulatory agents have generally been used for the treatment of immune dysregulation, as well as infections or cancers [6,7]. Anti-oxidants improved immune function and decreased mortality induced by infections, such as coronavirus disease [2,8]. However, deleterious adverse effects of the anti-oxidants or immunostimulatory agents have been reported [7,9]. Natural products exhibit various pharmacological properties, including anti-oxidants and immunity enhancement [10,11].

A prescription of traditional Korean medicine, Sambu-Tang (SBT), is extracted from *Panax ginseng* Meyer (*Araliaceae*) and *Aconitum carmichaeli* Debx (*Ranunculaceae*). *Panax ginseng* was reported to have diverse effects, such as improvement of immunity, memory, blood circulation, and anti-oxidation [12]. *Aconitum carmichaeli* was reported to have anti-osteoarthritis [13] and immunostimulatory effects [14]. SBT was reported to treat heart failure and septic shock [15,16]. Stigmasterol (Stig) was reported to be an active compound of both *Panax ginseng* and *Aconitum carmichaeli* [17,18]. We thus selected Stig, the shared component of *Panax ginseng* and *Aconitum carmichaeli,* as the active compound of SBT in this study. Stig exerts anti-oxidant [19], anti-nociceptive [20], anti-inflammatory [21], and anti-cancer effects [22]. However, the potential beneficial effects of SBT or Stig on immune dysregulation are currently unknown.

Macrophages actively modulate immune responses by releasing nitric oxide (NO) and cytokines, such as TNF-α and IL-6 [23]. Spleen is a peripheral immune organ where immune cells, including macrophages, monocytes, T cells, B cells, and neutrophils, function as immune effectors [24]. Cyclophosphamide (CTX) as an anti-cancer drug or an immunosuppressive agent has been used for the treatment of cancer or immune-mediated diseases [25]. Because CTX induces oxidative stress and has immunosuppressive properties, the CTX-induced immunosuppressive murine model has been extensively studied to establish the immunomodulatory effects of agents [26,27,28,29,30].

In the present study, we exploited the potential of SBT or Stig as an anti-oxidant and immunostimulatory agent using RAW264.7 macrophage cells and splenocytes. We conducted in vivo experiments in the established murine model of CTX-induced immunosuppression to provide evidence supporting optimal effects by the administration of SBT or Stig.

## 2. Materials and Methods

### 2.1. Preparation of SBT and Stig

SBT was prepared according to a traditional oriental medicine book (Jing Yue Quan Shu, written in 1624), as previously described, with a few modifications [31]. SBT was extracted from 40 g of *Panax ginseng* Meyer (dried root) and 40 g of *Aconitum carmichaeli* Debx (dried root) at a 1:1 ratio in 30% ethanol at 50 °C for 4 h with reference to previous reports [17,32,33]. The extract was evaporated under reduced pressure at 50 °C and then freeze-dried at −80 °C for 72 h. The lyophilized powder was dissolved in distilled water and further diluted with culture media (yield = 5%). Stig (purity ≥ 95%, Sigma-Aldrich Co., St. Louis, MO, USA) was dissolved in ethanol and further diluted with culture media (ethanol < 0.02%) [34].

### 2.2. High Performance Liquid Chromatography (HPLC) Analysis

**Figure 1:**
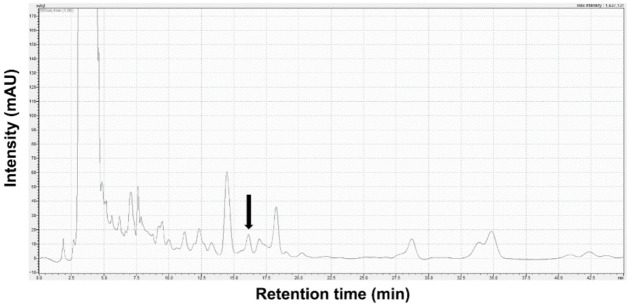
HPLC chromatogram for Stig analysis in SBT. Black arrow indicates Stig.

The identification of Stig in SBT was analyzed using Shimadzu 20A HPLC equipment combined with a UV detector (Shim-pack GIS 5 μm ODS 4.6 × 250 mm, Shimadzu Co., Kyoto, Japan). The isocratic elution mode was applied with a mobile phase consisting of 100% methanol flowing at a rate of 1.0 mL/min. The absorption wavelength selected was 205 nm. The injection volume was 30 µL. Stig was detected at a retention time of 16.12 min. The content of Stig in SBT was 3.96 ± 0.18 μg/g (Figure 1).

### 2.3. Cell Cultures

The RAW264.7 macrophage cell line was incubated in Dulbecco’s Modified Eagle’s Medium (Gibco BRL, Grand Island, NY, USA), supplemented with 10% fetal bovine serum (FBS, Neuromics, Edina, MN, USA), streptomycin (100 μg/mL)/penicillin (100 U/mL, Sigma-Aldrich Co, St. Louis, MO, USA) at 37 °C under humidified air with 5% CO_2_. Isolated spleens from BALB/c mice (Dae-Han Experimental Animal Center, Eumseong, Republic of Korea) were gently pressed and then forced through a 40 µm cell strainer in RPMI-1640 media (Gibco BRL, Grand Island, NY, USA). YAC-1 lymphoma cells were incubated in RPMI-1640 media supplemented with 10% FBS. RAW264.7 macrophage cells, splenocytes, and YAC-1 cells were incubated with SBT (1, 10, and 100 µg/mL), Stig (1 µg/mL), or lipopolysaccharide (LPS, 10 ng/mL, Sigma-Aldrich Co., St. Louis, MO, USA).

### 2.4. The 3-(4,5-Dimethylthiazol-2-yl)-2,5-Diphenyltetrazolium Bromide (MTT) Assay

Cell viability was analyzed by an MTT (Sigma-Aldrich Co., St. Louis, MO, USA) assay. RAW264.7 macrophage cells and splenocytes were received with various concentrations of SBT or Stig. Then, the supernatant was discarded, and an MTT solvent (5 mg/mL) was added. The supernatant was discarded, and dimethyl sulfoxide was added to dissolve the insoluble formazan product. The absorbances at 570 nm were obtained by a microplate reader (Molecular Devices, LLC., Sunnyvale, CA, USA).

### 2.5. Nitrite Production

The supernatants from RAW264.7 macrophage cells were collected and reacted with Griess reagent I and Griess reagent II. A NaNO_2_ standard curve was used to calculate nitrite concentrations. The nitrite concentrations were assessed at 540 nm by a microplate reader.

### 2.6. RNA Extraction and Quantitative Real-Time PCR (qRT-PCR)

Total RNA was extracted from RAW264.7 macrophage cells (1 × 10^6^) and splenocytes (1 × 10^7^) using an easy-BLUETM RNA extraction kit (iNtRON Biotech Inc., Seongnam, Korea). The products were reverse-transcribed into cDNA using a cDNA synthesis kit (Bioneer Corporation, Daejeon, Korea). A qRT-PCR (Applied Biosystems, Foster City, CA, USA) was conducted with Power SYBR Green Master Mix (Thermo Fisher Scientific, Waltham, MS, USA) according to the instructions from the manufacturer. The calculation of the relative expression of target genes is based on the use of an endogenous control, GAPDH. The following primers were used: inducible NO synthase (iNOS): 5’-CAT TGG AAG TGA AGC GTT TCG-3’ and 5’-CAG CTG GGC TGT ACA AAC CTT-3’; heme oxygenase-1 (HO-1): 5′-CTC CCT GTG TTT CCT TTC TCT C-3′ and 5′-GCT GCT GGT TTC AAA GTT CAG-3′; KI-67: 5′-CAT CAG CCC ATG ATT TTG CAA C-3′ and 5′-CTG CGA AGA GAG CAT CCA TC-3′; NCR1: 5′-ATG CTG CCA ACA CTC ACT G-3′ and 5′-GAT GTT CAC CGA GTT TCC ATT TG-3′; NKG2D: 5′-ACT CAG AGA TGA GCA AAT GCC-3′ and 5′-CAG GTT GAC TGG TAG TTA GTG C-3′; KLRD1: 5′-TCT AGG ATC ACT CGG TGG AGA-3′ and 5′-CAC TTG TCC AGG CAA ACA CAG-3′; CD49b: 5′-TGT CTG GCG TAT AAT GTT GGC-3′ and 5′-CTT GTG GGT TCG TAA GCT GCT-3; GAPDH: 5′-GGC AAA TTC AAC GGC ACA-3′ and 5′- GTT AGT GGG GTC TCG CTC CTG-3′.

### 2.7. Cell Lysis and Western Blotting

RAW264.7 macrophage cells (5 × 10^6^) and splenocytes (2 × 10^7^) were washed with ice-cold PBS and lysed with a RIPA Buffer (Sigma-Aldrich Co., St. Louis, MO, USA) supplemented with a protease inhibitor cocktail. Nuclear and cytosolic fractions were prepared, as previously described [35]. Total protein concentration was measured with a BCA protein assay kit (Thermo Fisher Scientific). The cellular lysates were subjected to 12% SDS-PAGE, followed by Western blot with antibodies against internal loading controls (GAPDH, actin, and poly (ADP-ribose) polymerase (PARP)), iNOS, HO-1, NF-κB, pIκBα, and horseradish peroxidase-conjugated secondary antibodies (Santa Cruz Biotechnology, Santa Cruz, CA, USA). The blots were probed using an enhanced chemiluminescence Western blot detection system (DoGenBio Co., Seoul, Korea), as instructed by the manufacturer.

### 2.8. Enzyme-Linked Immunosorbent Assay (ELISA) for Cytokines

The levels of granulocyte colony-stimulating factor (G-CSF, R&D Systems, Minneapolis, MN, USA), IFN-γ (BD Biosciences, San Jose, CA, USA), IL-12 (BD Biosciences), IL-2 (BD Biosciences, San Jose, CA, USA), IL-6 (BD Biosciences, San Jose, CA, USA), and TNF-α (BD Biosciences, San Jose, CA, USA) in cell-culture supernatants, serum, and spleen homogenates were measured by ELISA according to the manufacturer’s instructions, as previously described [36].

### 2.9. Immunofluorescence

Cells were fixed in 4% paraformaldehyde solution, permeabilized in 0.2% Triton X-100 and blocked in 5% bovine serum albumin at room temperature. Next, the cells were incubated in diluted antibodies, anti-NF-κB, anti-KI-67, anti-mouse IgG H&L (Alexa Fluor 647, Abcam, Cambridge, MA, USA), or anti-goat IgG H&L (Alexa Fluor 488, Abcam, Cambridge, MA, USA). The nuclei were labelled with Fluoroshield mounting medium with 4′,6-diamidino-2-phenylindole (DAPI). Images were observed using Zeiss LSM800 fluorescent confocal microscopy (Carl Zeiss, Oberkochen, Germany).

### 2.10. Measurement of Splenic Lymphocyte Proliferation

The splenocytes proliferation (1 × 10^4^) was determined with a bromodeoxyuridine (BrdU) cell proliferation assay kit, as instructed by the manufacturer (BioVision Inc., Milpitas, CA, USA).

### 2.11. Measurement of NK Cell Cytotoxicity

The target cells, the YAC-1 lymphoma cells, and the effector cells, splenocytes, were seeded into 24-well plates at a ratio of effector cells to target cells of 50:1 and 200:1. The cells were treated with SBT or Stig for 20 h, followed by a mixing of the culture supernatant with a lactic acid dehydrogenase (LDH) solution (DoGenBio Co., Seoul, Korea). The NK activity of effector cells was calculated as follows: cytotoxicity (%) = (experimental release − spontaneous release/maximum release − spontaneous release) × 100.

### 2.12. Mice and Experimental Design

Male BALB/c mice (6 weeks of age) obtained from Dae-Han Experimental Animal Center had free access to a standard diet and water. The in vivo study was designed according to the previous reports [28,29]. After a 7-day acclimatization period, the mice were randomly divided into five groups (*n* = 6). From day 1 to day 3, four groups of mice were intraperitoneally injected with CTX at 100 mg/kg per day, except the normal group. The CTX-injected mice were gavaged daily with phosphate-buffered saline (PBS), SBT (100 mg/kg), Stig (1 mg/kg), and Korean Red Ginseng (RG, 400 mg/kg) for 7 consecutive days from day 4 to day 10. The body weight gains were calculated as follows: body weight gains (g) = weight on day 10 − weight on day 0. The spleen index was calculated as follows: spleen index (mg/g) = organ weight/body weight. The superoxide dismutase (SOD) activity was assayed in spleen homogenate (BioVision Inc.). The mice used in this study were handled in accordance with the guidelines for the Care and Use of Laboratory Animals (the U.S. National Institutes of Health, publication No. 85-23. Revised 1985). All experimental procedures were approved by the Animal Care Committee of Kyung Hee University (Ethics NO. KHSASP-20-472).

### 2.13. Statistical Analysis

Statistical significances were analyzed by one-way analysis of variance with Tukey’s post hoc test (IBM SPSS software, Armonk, NY, USA). Statistical significance was considered at *p* < 0.05. The values show as mean ± standard error of the mean (SEM).

## 3. Results

### 3.1. SBT or Stig Enhances the Secretion of NO and Expression of HO-1 in RAW264.7 Macrophage Cells

**Figure 2:**
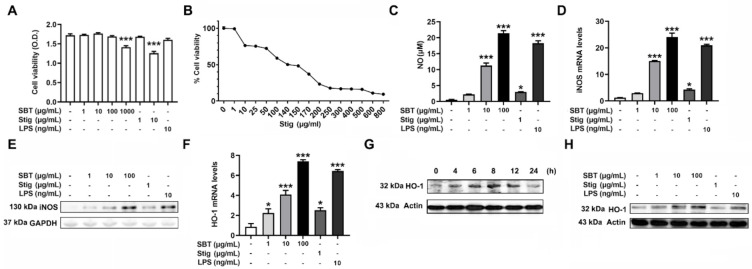
SBT or Stig enhances the levels of NO and HO-1 in RAW264.7 cells. (**A**) Cell viability was determined by an MTT assay after treatment with SBT, Stig, or LPS for 48 h. (**B**) IC50 was analyzed by an MTT assay after the treatment with Stig for 48 h. (**C**) The concentration of NO was determined by the Griess reactions after treatment with SBT, Stig, or LPS for 48 h. (**D**) The mRNA levels were determined by qRT-PCR after treatment with SBT, Stig, or LPS for 12 h. (**E**) The protein levels of iNOS were determined by Western blotting after treatment with SBT, Stig, or LPS for 24 h. GAPDH served as a loading control. (**F**) The mRNA levels were determined by qRT-PCR after treatment with SBT, Stig, or LPS for 6 h. * *p* < 0.05 and *** *p* < 0.001 vs. untreated group. (**G**) The protein levels of HO-1 were determined by Western blotting after the treatment with SBT (100 μg/mL). (**H**) The protein levels of HO-1 were determined by Western blotting after treatment with SBT, Stig, or LPS for 8 h. Actin served as a loading control.

First, an MTT assay was carried out to examine the cytotoxicity of SBT and Stig. SBT at 1000 μg/mL and Stig at 10 μg/mL exhibited the cytotoxicity (*p* < 0.05, Figure 2A). Thus, the concentrations of SBT were determined to be 1, 10, and 100 μg/mL lower than 1000 μg/mL. The concentration of Stig was determined to be 1 μg/mL lower than 10 μg/mL, with reference to a previous report [22]. The IC50 value of Stig against RAW264.7 macrophage cells was 140 μg/mL (Figure 2B). Activated macrophages release an effector molecule, NO, to inhibit pathogen replication [37]. In addition, NO acts as a powerful anti-oxidant [38]. Thus, we investigated whether SBT and Stig could up-regulate the NO pathways. As shown in Figure 2C, SBT or Stig significantly increased the secretion levels of NO (*p* < 0.05). SBT or Stig significantly increased the levels of mRNA expression of inducible NO synthase (iNOS) (*p* < 0.05, Figure 2D). The protein expression of iNOS was also increased by SBT or Stig (Figure 2E). In addition, SBT or Stig significantly increased the levels of mRNA expression of a phase 2 detoxifying enzyme, HO-1, which has an important antioxidant effect [39] (*p* < 0.05, Figure 2F). The protein expression level of HO-1 reached the maximum value at 8 h after treatment with SBT and then decreased (Figure 2G). Thus, the cells were treated with SBT or Stig for 8 h, and the regulatory effects on the protein expression level of HO-1 were analyzed. As expected, SBT or Stig increased the protein expression levels of HO-1 (Figure 2H). LPS as a positive control [40] also increased the levels of NO, iNOS, and HO-1 (Figure 2C–F,H).

### 3.2. SBT or Stig Enhances the Secretion of Immunostimulatory Cytokines from RAW264.7 Macrophage Cells

**Figure 3:**
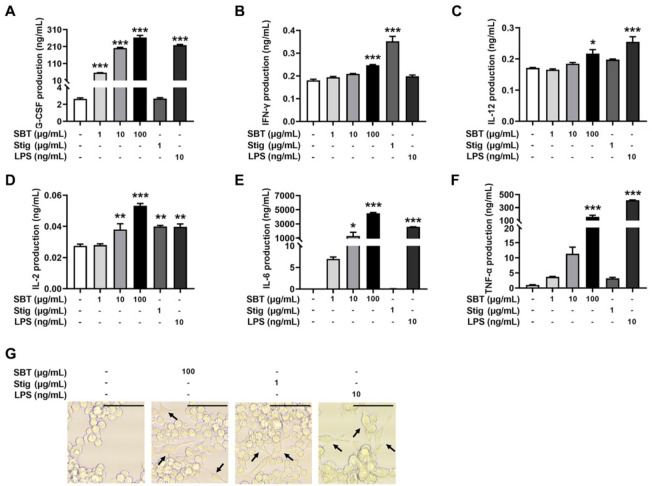
SBT or Stig enhances the secretion of immunostimulatory cytokines from RAW264.7 cells. (**A**–**F**) Each concentration was determined by ELISA after treatment with SBT, Stig, or LPS for 24 h. (**G**) Morphological alteration of RAW264.7 cells treated with SBT, Stig, or LPS for 24 h was observed under a light microscope (scale bar = 50 μm). Arrows indicate pseudopods. * *p* < 0.05, ** *p* < 0.01, and *** *p* < 0.001 vs. untreated group.

To evaluate whether the treatment with SBT and Stig could enhance the secretion of immunostimulatory cytokines from macrophages, SBT and Stig were treated in RAW264.7 macrophage cells. Figure 3A–F shows that the production levels of G-CSF, IFN-γ, IL-12, IL-2, IL-6, and TNF-α were increased following SBT treatment (*p* < 0.05). Stig significantly increased the levels of IFN-γ and IL-2 (*p* < 0.05, Figure 3B, D). LPS also significantly increased the production levels of G-CSF, IL-12, IL-2, IL-6 and TNF-α (*p* < 0.05, Figure 3A–F). Next, we observed the morphological alterations associated with macrophage activation in response to SBT and Stig. As shown in Figure 3G, most normal cells had a circular morphology, whereas the morphologies in the presence of SBT or Stig were changed irregularly with spreading and pseudopodia formation similar to LPS-treated cells (Figure 3G), indicating the enhanced functions of RAW264.7 macrophage cells by treatment with SBT or Stig [41,42].

### 3.3. SBT or Stig Up-Regulates NF-κB Activation in RAW264.7 Macrophage Cells

**Figure 4:**
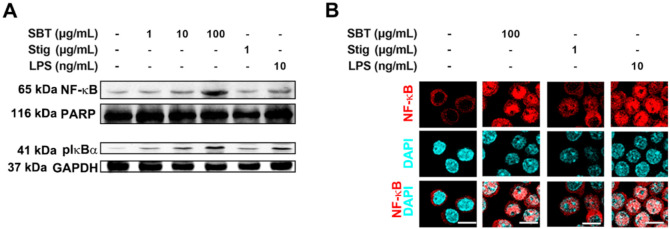
SBT or Stig enhances NF-κB activation in RAW264.7 cells. (**A**) The protein levels of nuclear NF-κB and cytoplasmic pIκBα were determined by Western blotting after the treatment with SBT, Stig, or LPS for 2 h. PARP and GAPDH served as loading controls. (**B**) The translocation of NF-κB (red) into nucleus was determined after treatment with SBT, Stig, or LPS for 2 h under a fluorescence microscopy (scale bar = 10 μm). The nuclei were labelled with DAPI (blue).

In order to dissect the mechanisms underlying immune-stimulation following the treatment of SBT and Stig, we investigated whether SBT and Stig could increase NF-κB activation in RAW264.7 macrophage cells. Western blotting indicated that SBT or Stig remarkably increased the protein levels of nuclear NF-κB and cytoplasmic phosphorylated IκBα (pIκBα) in RAW264.7 macrophage cells (Figure 4A). The increased expression levels of NF-κB in the nucleus were visualized by immunofluorescence microscopy (Figure 4B).

### 3.4. SBT or Stig Enhances the Secretion of Immunostimulatory Cytokines from Splenocytes

**Figure 5:**
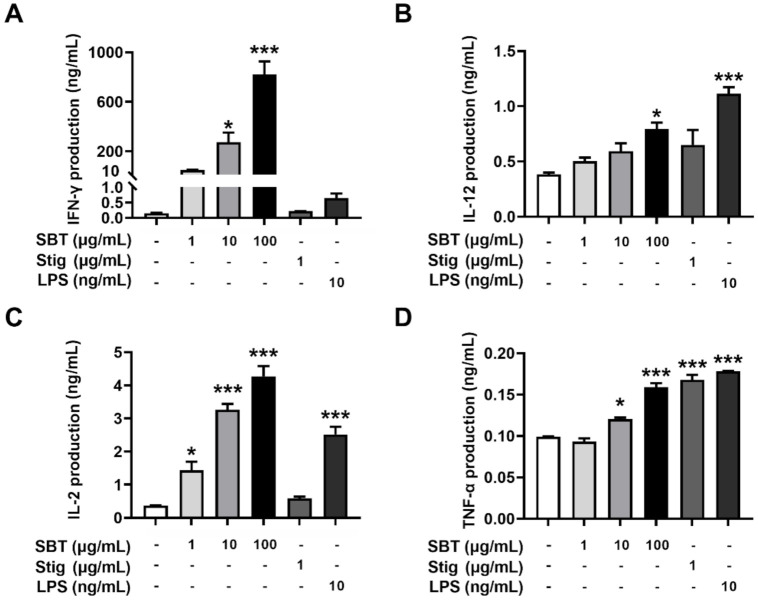
SBT or Stig enhances the secretion of immunostimulatory cytokines from splenocytes. (**A**–**D**) Each concentration was determined by ELISA after treatment with SBT, Stig, or LPS for 72 h. * *p* < 0.05 and *** *p* < 0.001 vs. untreated group.

We further evaluated whether SBT and Stig could enhance the secretion of immunostimulatory cytokines from splenocytes. As expected, SBT significantly increased the secretion levels of IFN-γ, IL-12, IL-2, and TNF-α (*p* < 0.05, Figure 5). Stig significantly increased the secretion levels of TNF-α (*p* < 0.05, Figure 5D). LPS also significantly increased IL-12, IL-2, and TNF-α levels.

### 3.5. SBT or Stig Promotes Splenic Lymphocyte Proliferation

**Figure 6:**
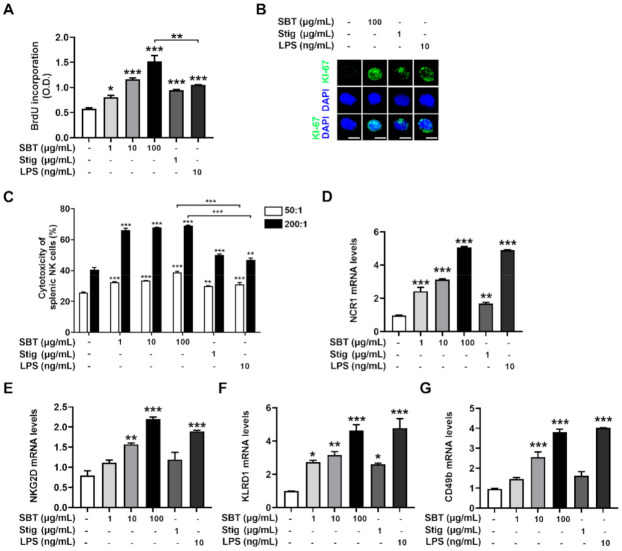
SBT or Stig promotes splenic lymphocyte proliferation and splenic NK cell cytotoxic activity. (**A**) Cell proliferation was analyzed with a BrdU incorporation assay kit after treatment with SBT, Stig, or LPS for 48 h. (**B**) KI-67 staining (green) and DNA counterstaining with DAPI (blue) were determined after treatment with SBT, Stig, or LPS for 48 h under a fluorescence microscopy (scale bar = 2 μm). (**C**) The cytotoxic effect of NK cells towards target cells was measured with an LDH assay kit after the treatment with SBT, Stig, or LPS for 20 h, 50:1 and 200:1, a ratio of effector cells to target cells. (**D**–**G**) The mRNA levels were determined by qRT-PCR after treatment with SBT, Stig, or LPS for 8 h. * *p* < 0.05, ** *p* < 0.01, and *** *p* < 0.001 vs. untreated group.

Splenocytes proliferation elevates the expression of cytokines, potentially improving cell-mediated immune responses and resulting in immunity-enhancement [43]. The incorporation of BrdU into proliferating cells has been used as a measure of cell proliferation [44]. Thus, we evaluated whether SBT and Stig could promote splenocytes proliferation by analyzing the incorporation of BrdU. SBT or Stig significantly promoted the splenic lymphocyte proliferation (*p* < 0.05, Figure 6A). Interestingly, at least in this part, the splenic lymphocyte proliferation level of SBT was higher than that of LPS (*p* < 0.05). We further analyzed the expression levels of KI-67, which is a marker of cell proliferation [45]. The expression levels of KI-67 increased by the treatment with SBT or Stig were visualized in the nucleus by immunofluorescence microscopy (Figure 6B).

### 3.6. SBT or Stig Enhances Splenic Nature Killer (NK) Cell Cytotoxic Activity

NK cells as an innate immune cell have direct cytotoxic activity against tumor cells, release several cytokines, and regulate the function of innate and adaptive immune cells [46]. The cytotoxic activity of NK cells against YAC-1 targets is analyzed by measuring the LDH derived from lysed target cells in the medium following incubation of effector cells with target cells [27,47,48]. Thus, we evaluated whether SBT and Stig could increase splenic NK cell cytotoxic activity by conducting an LDH assay. We first confirmed that neither SBT nor Stig had splenocyte toxicity (Supplementary Figure S1). SBT or Stig significantly improved the splenic NK cell cytotoxic activity (*p* < 0.05, Figure 6C). Interestingly, at least in this part, the NK cell cytotoxic activities of SBT were higher than those of LPS (*p* < 0.05). NK cells are identified by the presence of NCR1, NKG2D, KLRD1, and CD49b, which are common NK cell markers [49,50]. SBT or Stig significantly increased the mRNA expression levels of NCR1, NKG2D, KLRD1, and CD49b in splenocytes, suggesting an increase in NK cell population (*p* < 0.05, Figure 6D–G).

### 3.7. SBT or Stig Enhances the Levels of Immunostimulatory Cytokines in the Serum of CTX-Induced Immunosuppressed Mice

**Figure 7:**
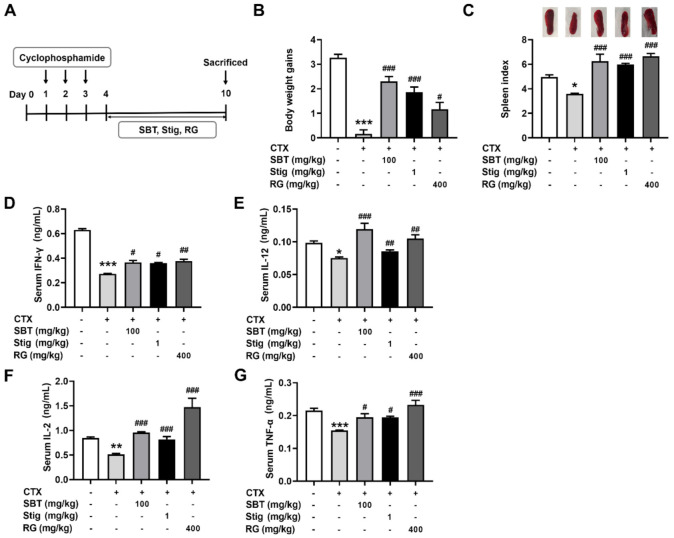
SBT or Stig enhances the levels of immunostimulatory cytokines in the serum of CTX-induced immunosuppressed mice. (**A**) Schematic diagram of the experimental protocol. (**B**) Body weight gains, (upper panel, **C**) representative pictures of the spleen, and (lower panel, **C**) spleen index were analyzed as described in Materials and Methods. (**D**–**G**) Each concentration in the serum was determined by ELISA. * *p* < 0.05, ** *p* < 0.01, and *** *p* < 0.001 vs the normal group. ^#^ *p* < 0.05, ^##^ *p* < 0.01, and ^###^ *p* < 0.001 vs. the CTX control group.

We evaluated a regulatory effect of SBT and Stig on immunosuppression using an established murine model of CTX-induced immunosuppression (Figure 7A). Each dose was determined by an MTT assay (Figure 2A) with reference to the previous reports [18,21,51,52,53,54,55]. As shown in Figure 7B,C, the CTX control group showed remarkably reduced body weight gains and spleen index compared to the normal group (*p* < 0.05). SBT or Stig significantly increased the body weight gains and spleen index reduced by CTX (*p* < 0.05). In addition, the levels of IFN-γ, IL-12, IL-2, and TNF-α in the serum of the SBT-treated mice and Stig-treated mice were significantly higher than those in the CTX control group (*p* < 0.05, Figure 7D–G). RG was used as a positive control in the CTX-induced immunosuppressed mouse model [56].

### 3.8. SBT or Stig Restores the Spleen Functions in the CTX-Induced Immunosuppressed Mice

**Figure 8:**
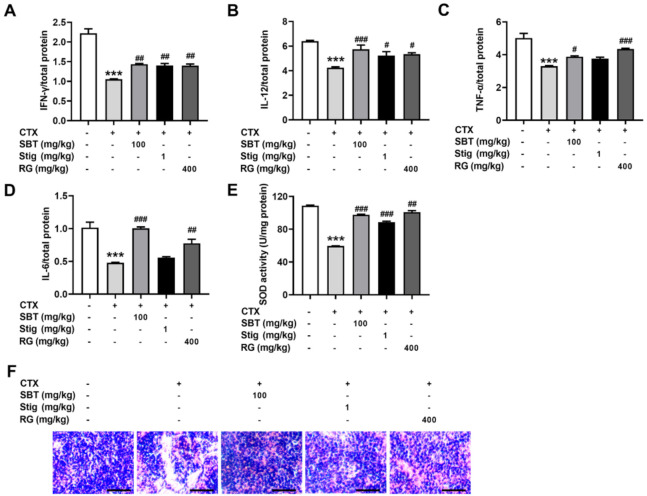
SBT or Stig restores the spleen functions in the CTX-induced immunosuppressed mice. (**A**–**D**) Each concentration in the spleen tissues was determined by ELISA. (**E**) The SOD activity in the spleen tissues was analyzed with an SOD assay kit (**F**) Representative pictures of H&E stained-spleen tissues were observed under a light microscope (scale bar = 40 μm). *** *p* < 0.001 vs. the normal group. ^#^ *p* < 0.05, ^##^ *p* < 0.01, and ^###^ *p* < 0.001 vs. the CTX control group.

Finally, we investigated whether SBT and Stig could restore the spleen functions in the CTX-induced immunosuppressed mice. CTX exhibited a reduction in the levels of IFN-γ, IL-12, TNF-α, and IL-6 in the spleen, while the administration of SBT exhibited significant increases in the levels of IFN-γ, IL-12, TNF-α, and IL-6 (*p* < 0.05, Figure 8A–D). Stig significantly increased the levels of IFN-γ and IL-12 (*p* < 0.05, Figure 8A,B). In addition, SBT or Stig significantly increased the activity of an antioxidant enzyme, SOD, in the spleen (*p* < 0.05, Figure 8E). H&E stained-spleen tissues showed that the arrangement of splenocytes in the CTX control group was sparse and irregular, with necrotic areas, but the SBT or Stig-treated CTX group displayed relative tightness with fewer necrotic areas compared to the CTX control group (Figure 8F).

## 4. Discussion

In the present study, we identified, for the first time, the potential of SBT and Stig to enhance immune functions, by studying the effects on macrophages and splenocytes under CTX-induced immunosuppressive conditions. Our results revealed that SBT or Stig increased the secretion levels of NO and immunostimulatory cytokines from macrophages or splenocytes. These effects were associated with a significant improvement of NF-κB activation. In addition, SBT or Stig increased HO-1 expression levels. SBT or Stig also enhanced splenic lymphocyte proliferation and splenic NK cell cytotoxic activity. SBT or Stig prevented immunosuppressive responses in mice.

The activated macrophages produce a variety of immunomodulators, such as NO [57]. NO, as an important component of the immune system, exhibits anti-oxidant, anti-pathogen, and anti-tumor activities [38,58]. NO is produced as a primary product of iNOS in macrophages through activation of the NF-κB pathway [38,59]. Oxidative stress was enhanced in iNOS-deficient mice after traumatic brain injury [58]. NF-κB knockout mice revealed severe defects in immune function [60]. In the present study, the enhancement of release levels of NO and expression levels of iNOS by SBT or Stig may result from the increase in NF-κB activation. An anti-oxidant, vitamin D, increased NO levels and enhanced anti-oxidant defenses [61]. Anti-oxidants, such as vitamin C and selenium, improved immune responses [62]. In addition, we found that SBT or Stig increased the HO-1 expression and SOD activity. Thus, it is conceivable that SBT or Stig as an anti-oxidant exerts immunity-enhancing effects.

In the immune response, the cytokine network is complicatedly connected between immune cells [63]. The activated macrophages release various cytokines, such as G-CSF, IFN-γ, IL-12, and TNF-α as major components of innate immune responses [64,65]. G-CSF as a hematopoietic growth factor promotes the functional maturation of neutrophils and plays an essential role in host defense against infection [65]. IFN-γ has an important role in recognizing and eliminating pathogens, and increasing the efficiency of the immune system [66]. IL-12, a well-known IFN-γ inducer, participates in host defenses against various microbial pathogens [5]. IL-2 as an immune growth factor plays a critical role in sustaining T cell responses [67]. IL-6, if not excessively synthesized, contributes to host defense through activation of immune responses when homeostasis is disrupted by infections [68]. As a proinflammatory cytokine, dysregulated excessive synthesis of IL-6 has a pathological effect on inflammatory responses. This might stem from different cellular environments, signaling pathways, and doses of IL-6 [68,69,70]. G-CSF therapy reduced the incidence of infections in patients with neutropenia [71]. When immune-supportive IFN-γ was added to antimicrobial therapy, the general condition of patient suffering from infection was improved [72]. TNF-α treatment played a protective role in the acute stage of viral myocarditis in mice [73]. The present study showed that SBT augmented the release levels of G-CSF, IFN-γ, IL-12, IL-2, IL-6, and TNF-α from macrophages, which is in concordance with studies indicating their potential as immunity-enhancing agents by increasing the levels of immunostimulatory cytokines from macrophages [29,74]. However, Stig only increased IFN-γ and IL-2 levels from macrophages. This result suggests that the effect of SBT is potentially related to the various compounds present in SBT rather Stig alone.

The activated NK cells contribute to effective immune reactions against infections and cancers via cellular cytotoxicity and cytokine production [75]. NK cells deficiency was observed in patients with recurrent viral infections and cancer [76]. Monmai et al. [27] reported that macromolecules isolated from seaweeds improve the NK cell activity, presenting it as a potent material for enhancing immunity. Becker et al. [77] reported that NK cells have been used in several clinical trials as an immunotherapy, and stimulation of NK cell activity by IL-12 and IL-2 enhances the immunotherapy of NK cells. In the present study, SBT or Stig remarkably elevated splenic NK cell cytotoxic activity, increasing the release levels of IL-12 and IL-2 from splenocytes. Thus, we speculate that SBT or Stig may have applications for NK cell-based immunotherapy.

We finally investigated whether SBT and Stig could be stable in the culture medium. The treatment with SBT or Stig did not affect the pH values of the culture medium (Supplementary Figure S2), indicating that there were no changes in conditions of the culture medium or stable conditions of the SBT or Stig-added medium. Furthermore, several reports have suggested that SBT and Stig exert beneficial effects in various long-term experiments [22,78,79,80,81,82,83]. Thus, we presuppose that SBT or Stig may be stably maintained in the culture medium.

CTX has immunosuppressive effects and is applied as an immunosuppressant [84]. In addition, several studies reported that the injection of single doses of CTX (250–400 mg/kg) and smaller divided doses induce tolerance against ovine erythrocytes, bovine gamma globulin, and equine gamma globulin in mice [84,85,86]. To confirm the immunosuppressive properties of CTX, we assessed body weight and serum TNF-α and IL-6 levels instead of tolerance against the ovine erythrocyte test because many researchers determined the immunosuppression by measuring body weight and cytokines, such as TNF-α and IL-6 [26,28,29,30]. We showed that the body weight and serum TNF-α and IL-6 levels were reduced on the 7th day after CTX injection, indicating immunosuppression by CTX (Supplementary Figure S3). Immunodeficiency-related diseases cause body weight loss [87]. Because immune cells in the spleen colonize and respond to immune responses, the spleen index reflects the strength of innate immune function [88]. In the present study, SBT or Stig increased the body weight gains and spleen index in the CTX-induced immunosuppressed mice and improved the immune function of mice, increasing the levels of immunostimulatory cytokines in the serum and spleen. These findings indicate that SBT or Stig counteracts the immunosuppressive effect of CTX. Despite significant advances in our understanding of the immunity-enhancing effects of SBT or Stig in the CTX-induced immunosuppressed model, further studies are needed to clarify the regulatory effects of immune responses to various antigens in the CTX-induced immunosuppressed model and the immunity-enhancing effects in various immunosuppressive models.

The beneficial effects of SBT and Stig were practically the same, with Stig having effects at a lower concentration than SBT. However, SBT enhanced the various immune-related factors, such as G-CSF, IL-12, IL-6, and TNF-α from macrophages and IFN-γ, IL-12, and IL-2 from splenocytes more so than Stig did. Ginsenoside Rg3-enriched RG or ginsenoside Re-enriched Korean Ginseng was reported to have better effects in various experimental models [89,90]. Thus, Stig-enriched SBT could be expected to have a better effect on immunity enhancement.

## 5. Conclusions

In summary, the results indicate that SBT or Stig-mediated immune-enhancement, characterized by increased levels of immunostimulatory molecules in macrophages and splenocytes, may restore immunosuppressive responses in mice. The HO-1 expression levels, splenic lymphocyte proliferation, and splenic NK cell cytotoxic activity were elevated by SBT or Stig. Therefore, we speculate that SBT or Stig may exert curative properties as a potential immunity-enhancing agent.

## Data Availability

Data is contained within the article and Supplementary Materials.

## Supplementary Materials

The following supporting information can be downloaded at: www.mdpi.com/article/10.3390/antiox11020199/s1, Figure S1: Neither SBT nor Stig has splenocyte toxicity, Figure S2: SBT or Stig does not affect pH of culture medium, Figure S3: CTX leads to immunosuppression.
