# Supplementary material for: SBT (Composed of Panax ginseng and Aconitum carmichaeli) and Stigmasterol Enhances Nitric Oxide Production and Exerts Curative Properties as a Potential Anti-Oxidant and Immunity-Enhancing Agent
Source: Antioxidants (Basel). 2022 Jan 20;11(2):199. doi: 10.3390/antiox11020199 (PMC8868359; doi:10.3390/antiox11020199)
Supplement: Supplementary file 1 [file antioxidants-11-00199-s001.zip › antioxidants-1563062-supplementary.pdf]

Supplementary Materials

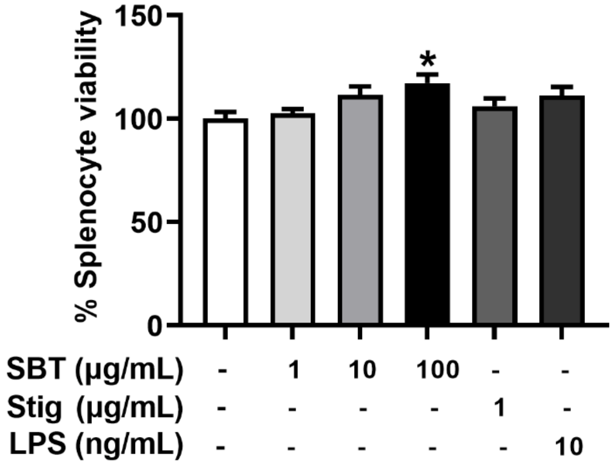

Figure S1. Neither SBT nor Stig has splenocyte toxicity. Splenocyte viability was determined by an MTT assay after the treatment with SBT, Stig, or LPS for 24 h. **p* < 0.05 vs untreated group.

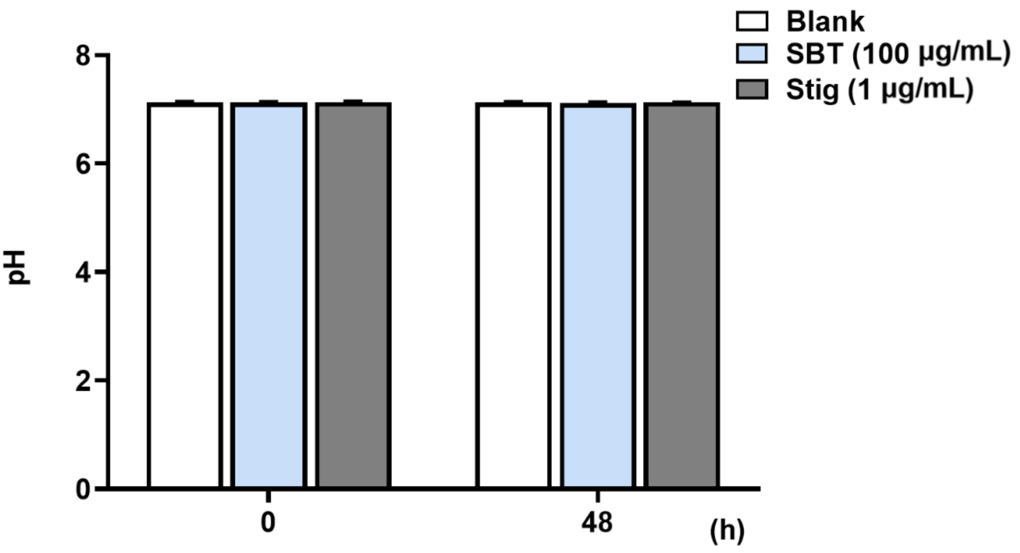

Figure S2. SBT or Stig does not affect pH of culture medium. The pH values were measured after treatment of SBT or Stig in culture medium at 37 °C for 48 h.

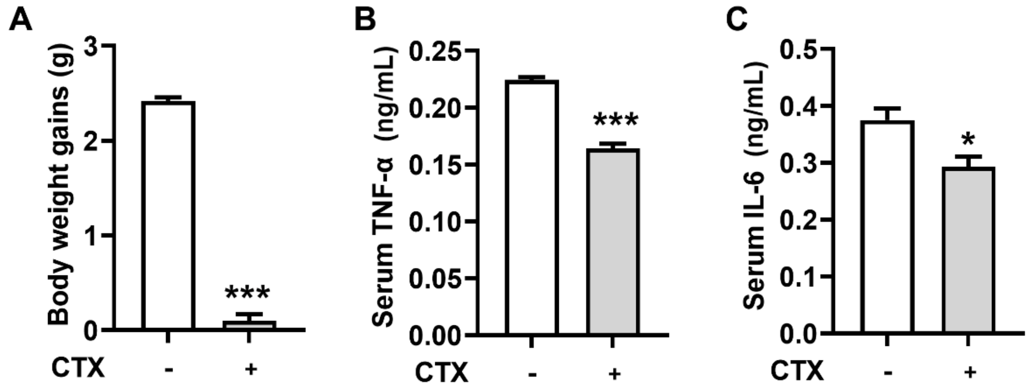

Figure S3. CTX leads to immunosuppression. From days 1–3, mice were intraperitoneally injected with CTX at 100 mg/kg per day except the normal group. From day 4 to day 10, both the normal group and the

CTX group were gavaged daily with PBS. (**A**) The body weight was analyzed as described in Materials and methods. (**B, C**) Each concentration in the serum was determined by ELISA. $^{*}p < 0.05$ and $^{***}p < 0.001$ vs the normal group. .
